# *BDNF* promoter methylation and genetic variation in late-life depression

**DOI:** 10.1038/tp.2015.114

**Published:** 2015-08-18

**Authors:** V Januar, M-L Ancelin, K Ritchie, R Saffery, J Ryan

**Affiliations:** 1 Cancer and Disease Epigenetics, Murdoch Childrens Research Institute, Royal Children's Hospital, Parkville, VIC, Australia; 2 Department of Paediatrics, University of Melbourne, Parkville, VIC, Australia; 3 Inserm U1061, Hopital La Colombiere & University Montpellier, Montpellier, France

## Abstract

The regulation of the brain-derived neurotrophic factor (BDNF) is important for depression pathophysiology and epigenetic regulation of the *BDNF* gene may be involved. This study investigated whether *BDNF* methylation is a marker of depression. One thousand and twenty-four participants were recruited as part of a longitudinal study of psychiatric disorders in general population elderly (age⩾65). Clinical levels of depression were assessed using the Mini International Neuropsychiatric Interview for the diagnosis of major depressive disorder according to the Diagnostic and Statistical Manual of Mental Disorder IV criteria, and the Centre for Epidemiologic Studies Depression Scale (CES-D) for assessment of moderate to severe depressive symptoms. Buccal DNA methylation at the two most widely studied *BDNF* promoters, I and IV, was investigated using the Sequenom MassARRAY platform that allows high-throughput investigation of methylation at individual CpG sites within defined genomic regions. In multivariate linear regression analyses adjusted for a range of participant characteristics including antidepressant use, depression at baseline, as well as chronic late-life depression over the 12-year follow-up, were associated with overall higher *BDNF* methylation levels, with two sites showing significant associations (promoter I, Δ mean=0.4%, *P*=0.0002; promoter IV, Δ mean=5.4%, *P*=0.021). Three single-nucleotide polymorphisms (*rs6265*, *rs7103411* and *rs908867*) were also found to modify the association between depression and promoter I methylation. As one of the largest epigenetic studies of depression, and the first investigating *BDNF* methylation in buccal tissue, our findings highlight the potential for buccal *BDNF* methylation to be a biomarker of depression.

## Introduction

As one of the most common psychiatric disorders, depression is a major public health problem, accounting for 40.5% of disability-adjusted life years worldwide.^1^ Despite this, depression is often under-recognized and undertreated, especially in geriatric populations.^2^ Compared with early-onset depression, late-life depression has a poorer prognosis and a higher illness burden, and is often a chronic disorder.^2^ Despite the abundance of symptom-based diagnostic tools, efforts to find objective biomarkers of the disorder have not been successful.

The heritability of major depression is estimated to be around 40%.^3, 4^ However despite intense efforts, few genetic variants have been identified.^5^ Among the potential candidates is the gene coding for brain-derived neurotrophic factor (*BDNF*), which promotes the proliferation, differentiation and survival of neurons and is crucial for neural plasticity and cognitive function.^6^ Lower circulating levels of BDNF have been observed in depressed patients compared with non-depressed individuals.^7^ Effective antidepressant treatments appear to increase circulating BDNF levels, and higher plasma BDNF may predict better antidepressant response.^8^ Several single-nucleotide polymorphisms (SNPs) in the *BDNF* gene have also been associated with the risk of major depression^9^ and the regulation of serotonin.^10^

Mounting evidence implicates epigenetic processes as driving the disrupted gene expression often observed in psychiatric disorders.^11^ Differential DNA methylation of several genes including *BDNF* has been reported in the blood of depressed individuals and in post-mortem brain tissue.^12^ However, these studies remain inconsistent, due to predominantly small sample size (often <100), as well as heterogeneity in participant characteristics, tissue types and diagnostic criteria. Not all studies have considered age, gender and ethnicity, which influence epigenomic profiles;^13, 14, 15^ antidepressant use and alcohol consumption, which are linked to both depression and epigenetic modifications, have also rarely been considered. Furthermore, despite the critical role of underlying genetic variation in determining the methylation status of many genomic loci,^16, 17^ only two studies of *BDNF* have considered both epigenetic and genetic variation, albeit at just a single SNP.^18, 19^ Thus the role of *BDNF* methylation in depression remains inconclusive and further large studies are needed.

We investigated *BDNF* methylation levels at two CpG islands within promoters I and IV, using DNA derived from buccal tissue, and determined whether there was an association with clinical levels of depression at baseline, as well as chronic depression. Analyses were adjusted for potential confounders, including antidepressant medication, and considered the potential role of genetic variation in modifying these associations.

## Materials and methods

### Participants

Participants were randomly recruited from electoral rolls as part of the ESPRIT study, a longitudinal French population study of psychiatric disorders.^20^ Participants were eligible if they were 65 years old or over, non-institutionalized and living in the Montpellier region at the time. They responded to standardized questionnaires and underwent extensive clinical assessments at their inclusion and at each follow-up wave (after 2, 4, 7, 10 and 12 years). Ethics approval was given by the regional ethics committee (Ethical Committee of University Hospital of Kremlin-Bicêtre, France). All participants provided written informed consent.

The diagnosis of current and past major depressive disorder (MDD) was performed by trained psychiatric nurses and psychologists according to Diagnostic and Statistical Manual of Mental Disorders-IV criteria and using the Mini International Neuropsychiatric Interview (MINI). The MINI is a standardized and structured diagnostic examination validated within the general population setting.^21^ Participants identified as having current MDD were reviewed further by a panel of three psychiatrists and a psychologist, with knowledge of the participants' medication and medical history, to validate the preliminary diagnosis. Severity of depressive symptoms was assessed using the Center for Epidemiologic Studies Depression (CES-D) scale. A score of 16 and above is widely regarded as a threshold for moderate to severe depression.^22, 23^ Late-life depression was thus defined here as CES-D⩾16 or current MDD. Participants were also classified according to the chronicity of depression over follow-up (MDD or CES-D⩾16 on three or more occasions across the study period).

The standardized interview included information regarding the demographic background, lifestyle and dietary habits, physical health, medical history and disabilities of participants collected through face-to-face interviews, and clinical examinations were conducted (Table 1). Participants with dementia were excluded from the study due to the likely impact on the results of other neuropsychiatric assessments and questionnaire responses.

Of the 2199 non-demented elderly recruited for the ESPRIT study, 1146 provided buccal samples for DNA extraction. Of these, 122 participants were not included in this study due to insufficient or poor quality DNA (*n*=112) or did not undergo assessment for depression (*n*=10). Compared with the participants included in the analysis (*n*=1024), those excluded had a lower educational level, were older, more likely to have cognitive dysfunction, comorbidity disease and depression, as well as to use antidepressants (*P*<0.001 for all comparisons).

### Genotyping

DNA was extracted from buccal tissue obtained around the fourth wave of follow-up using methods as described elsewhere^24^ and stored at −80 °C. *BDNF* genotyping was performed by KBiosciences (Middlesex, UK) using the KBioscience Competitive Allele-Specific PCR SNP genotyping system (KASPar).^25^ Genotype data were obtained for seven *BDNF* polymorphisms: *rs6265*, *rs11030101*, *rs28722151*, *rs7103411*, *rs962369*, *rs908867* and *rs1491850*, selected to represent variation across the entire gene and including variants previously associated with depression (Supplementary Figure S1).^26, 27^ *χ*^2^-tests were used to compare the distribution of genotypes with those predicted under Hardy–Weinberg equilibrium. Linkage disequilibrium between the SNPs was calculated using Haploview version 4.2 (Supplementary Figure S2).^28^

### Selection of genomic regions for methylation analysis

The human *BDNF* gene is comprised of 11 exons, 9 of which encode alternative first exons, each regulated by separate promoters that facilitate differential regulation of the *BDNF* gene.^29^ Two assays covering *BDNF* promoters I and IV were investigated, as these promoters have been the most widely implicated in the context of behavioural epigenetics.^30^ Promoter IV, in particular, has been found to have a dominant role in the epigenetic regulation of *BDNF* expression.^31, 32, 33^

Methylation assays were designed using Epidesigner software (http://www.epidesigner.com/) and visualized using the University of California, Santa Cruz (UCSC) genome browser. Assays cover the regions chr11:27 744 025-27 744 279 (promoter I) and chr11:27 723 096-27 723 467 (promoter IV) on the UCSC h19 assembly (Supplementary Figure S1; Supplementary Tables S1–3). A total of 11 CpG units were measured across promoter I, corresponding to 16 CpG sites (Supplementary Figure S1). For promoter IV, 7 CpG units were investigated, corresponding to 11 sites (Supplementary Table S2).

### Methylation analysis by Sequenom MassARRAY

Genomic DNA (500 ng) was bisulphite-converted using EZ-96 DNA Methylation-Lightning MagPrep (Irvin, CA, USA)^34^ and 25 ng used for subsequent PCR. As PCR is known to be the most variable step in methylation analysis, samples were PCR amplified and assayed in triplicate.^35^ DNA methylation was quantified using Sequenom MassARRAY (San Diego, CA, USA)^36^ and methylation ratios calculated using EpiTyper software (Sequenom, San Diego, CA, v.1.2). Methylation data for promoter I were obtained for all 1024 participants, whereas 312 participants were missing data for *BDNF* promoter IV, which was a longer assay and more troublesome with samples that had lower DNA quality (thus *n*=712; 183 with depression and 529 non-depressed). Participants missing promoter IV data were not significantly different from the overall population (*P*>0.05 for all comparisons).

### Data quality control and statistical analysis

Statistical analysis was performed using Stata 13 (StataCorp, College Station, TX, USA). There have been no previous studies measuring *BDNF* methylation in buccal tissue, thus we could not estimate effect sizes and subsequently study power. However, our sample size was considerably larger than the vast majority of studies investigating methylation levels in depression, suggesting that it was sufficiently powdered to detect an association if one indeed exists.

Mean methylation levels from three technical replicates were used, after discarding any outlying values (deviation of ±10% methylation from the median).^37^ Raw methylation values were log-transformed to normalise the data. If raw methylation was 0, an insignificant value (0.0001) was added to avoid undefined log-transformed results. Log-transformed values were used for subsequent statistical calculations.^38^ Univariate analysis (two-sided *χ*^2^-test and *t*-tests as appropriate) was performed to detect potential associations between population characteristics with depression and methylation levels. Multivariate linear regression analysis of the association between depression and methylation levels was performed, incorporating potential confounding factors to ensure they did not alter the association. Both baseline depression and chronic depression were investigated.

Results were stratified according to genotype when there was evidence that genotype modified the depression–methylation association (that is, a significant interaction term in the regression models). Sensitivity analysis excluded participants treated with antidepressants (*n*=40).

## Results

### Association between depression and methylation

**Figure 1:**
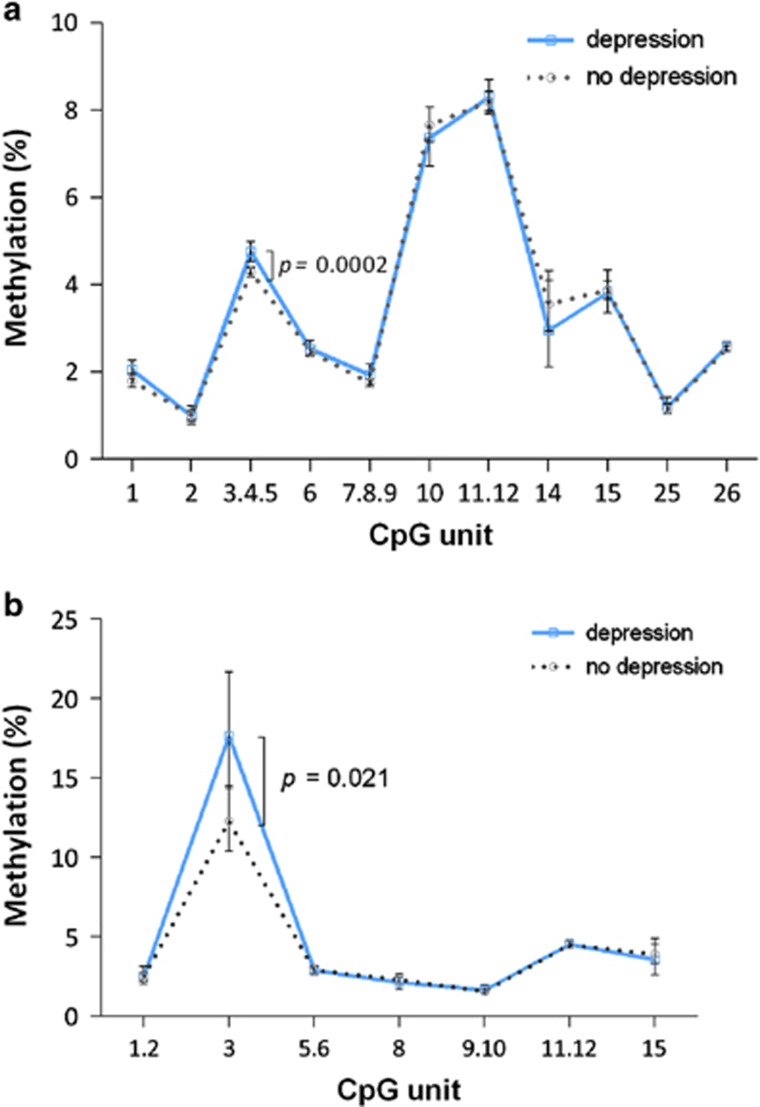
(**a**) Comparison of *BDNF* promoter I methylation between depressed and non-depressed individuals. Data are presented as the geometric mean methylation (%)±95% confidence interval of study participants for individual CpG units. *P*-values calculated from the Student's *t*-test (*n*=1024, except for CpG 14 with *n*=219 depressed, *n*=628 non-depressed). (**b**) Comparison of *BDNF* promoter IV methylation between depressed and non-depressed individuals. Data are presented as the geometric mean methylation (%)±95% confidence interval of study participants for individual CpG units. *P*-values calculated from the Student's *t*-test (*n*=712, except for CpG 3 with *n*=519 non-depressed, *n*=178 depressed).

Characteristics of the participants according to their depression status are shown in Table 1. In unadjusted linear regression analysis, depression was associated with a significantly higher level of *BDNF* promoter I methylation at CpG unit 3.4.5, with an effect size (Δ mean methylation) of 0.4%, *P*=0.0002 (Figure 1a). There was also a trend for increased methylation in CpG analytic units 1 (Δ=0.25%, *P*=0.097) and 7.8.9 (Δ=0.19%, *P*=0.074) (Supplementary Table S4). Furthermore, participants with depression had higher methylation levels at CpG site 3 of promoter IV (Δ=5.4%, *P*=0.021, Figure 1b). No significant differences in average methylation across the entire *BDNF* promoter I or IV assays were found between depressed and non-depressed individuals. The same associations were found in sensitivity analysis excluding users of antidepressant treatment (CpG 3.4.5 promoter I, Δ=0.45%, *P*=0.0007; CpG site 3 promoter IV, Δ=4.8%, *P*=0.050).

After adjustment for age, sex and antidepressant use, methylation of CpG unit 3.4.5 in *BDNF* promoter I (*β*=0.094, s.e.=0.029 and *P*=0.001) and CpG 3 in promoter IV (*β*=0.31, s.e.=0.14 and *P*=0.025) remained significantly associated with depression (Supplementary Table S5). The latter, however, did reduce in significance after additional adjustment for functional impairment (*β*=0.24, s.e.=0.13 and *P*=0.067). None of the other covariates listed in Table 1 including physical health factors and cognitive function, influenced the findings, suggesting that the differences observed were not driven by these other measures. There were no significant sex or antidepressant interactions in the final multivariate models either.

### Association between depression and methylation, modified by BDNF genotype

**Figure 2:**
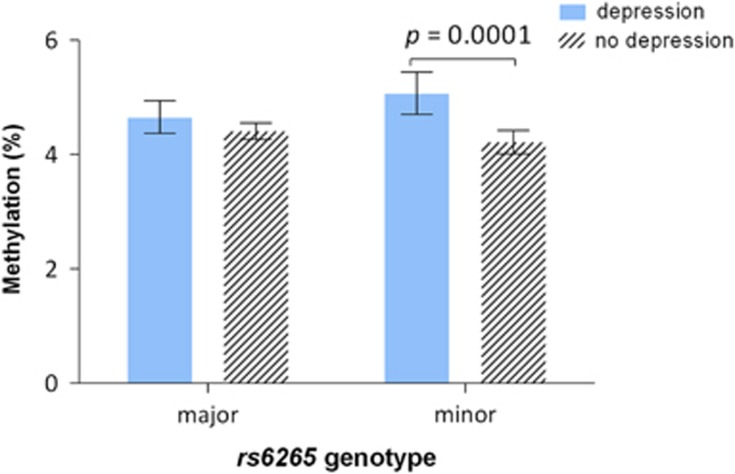
Comparison of *BDNF* promoter I methylation at CpG unit 3.4.5 in depressed and non-depressed individuals, stratified according to the presence of the *rs6265* minor allele. Data are presented as the geometric mean methylation (%)±95% CI. *P*-values calculated from the Student's *t*-test (*n*=584 major allele homozygotes, 371 heterozygotes and minor allele homozygotes). CI, confidence interval.

The frequencies of the *BDNF* SNPs (Table 2) were not significantly different from those predicted under Hardy–Weinberg equilibrium (*P*>0.14 for all SNPs). None of these SNPs were associated with average *BDNF* methylation across promoters I and IV; however, three were found to modify the previously observed association between depression and CpG unit 3.4.5 methylation levels (*P*-values for interaction term: *rs6265*, *P*=0.022, *rs7103411*, *P*=0.023 and *rs908867*, *P*=0.094). After stratification by allele, depression was found to be associated with higher methylation levels for the carriers of the minor allele of *rs6265* (Δ mean=0.9%, *P*=0.0001, Figure 2) and of *rs7103411* (Δ mean=0.8%, *P*=0.0002, Supplementary Figure S3), whereas for *rs908867* only major homozygotes showed a significant depression–methylation association (Δ mean=0.4%, *P*=0.0006, Supplementary Figure S4). For CpG analytical unit 7.8.9 of *BDNF* promoter I, a trend association of increased methylation with depression was observed as well as a modifying effect by two polymorphisms (*P*-values for interaction term: *rs908867*, *P*=0.046; *rs962369*, *P*=0.004). However, in stratified analysis, there were no significant associations between depression and methylation (data not shown).

### Chronic depression over follow-up

**Figure 3:**
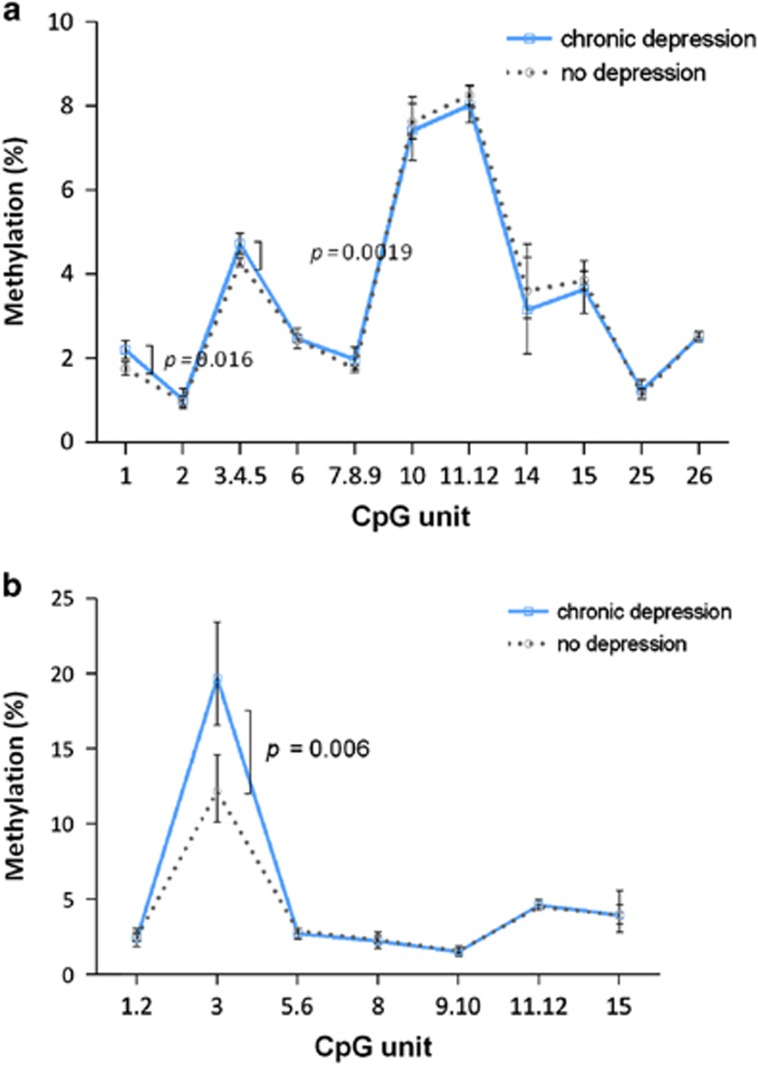
(**a**) Comparison of *BDNF* promoter I methylation between chronically depressed individuals and those without depression. Data are presented as the geometric mean methylation (%)±95% CI. *P*-values calculated from the Student's *t*-test (*n*=712 no depression, 185 chronic depression, except for CpG 14 with *n*=161 missing samples). Those with intermittent depression (*n*=127) were excluded from analysis. (**b**) Comparison of *BDNF* promoter IV methylation between chronically depressed individuals and controls. Data are presented as the geometric mean methylation (%)±95% CI. *P*-values calculated from the Student's *t*-test (*n*=488 non-depressed, 138 chronic depressed, except for CpG 3 with *n*=117 missing samples). Those with intermittent depression were excluded from analysis. CI, confidence interval.

Of the 1024 participants, 18% had chronic depression, that is, were depressed at three or more of the assessments. When we compared *BDNF* methylation between individuals with chronic depression (*n*=185) and those free of depression (*n*=712), the same pattern of association was observed as previously (Supplementary Table S4; Figure 3). The participants with chronic depression showed increased methylation at CpG 3.4.5 of promoter I (Δ mean=0.44%, *P*=0.0019) and CpG 3 promoter IV (Δ mean=7.52%, *P*=0.0061), which was slightly stronger than that with baseline depression. In addition, CpG 1 of promoter I showed significant elevated methylation with chronic depression (Δ=0.44%, *P*=0.016), and there was a similar trend for analytical unit 7.8.9 (Δ mean=0.22%, *P*=0.064). All of the associations remained significant after multi-adjustment.

## Discussion

We believe this is the largest study to investigate the role of *BDNF* epigenetics in depression and the first study to examine the relationship between *BDNF* methylation and depression using buccal-derived DNA. Furthermore, unlike previous studies, we included genetic variation across the *BDNF* gene as a potential modifier of the association between depression and methylation levels. We have shown that late-life depression is associated with elevated *BDNF* methylation of specific CpG sites within promoters I and IV, with all associations remaining after adjustment for a range of covariates. Similar associations were found with prevalent and chronic depression, and these effects were not driven by antidepressant treatment.

Promoter hypermethylation generally leads to reduced gene expression.^39^ Our finding of elevated *BDNF* promoter methylation associated with depression strongly supports observations of reduced BDNF levels in the plasma and post-mortem hippocampus of depressed individuals.^7, 8, 39, 40^ Decreased BDNF may relate to the reduced function of *BDNF* gene in promoting neural growth and repair in depression.^41^

### Comparison with previous findings

To our knowledge, no other study has investigated buccal *BDNF* methylation in the context of depression, and as methylation profiles can be tissue specific, this renders direct comparisons difficult. However, our findings support and extend previous results that have principally used blood samples, indicating elevated *BDNF* promoter methylation in depression.^18, 19, 42^

Four studies focusing on *BDNF* promoter I methylation in blood reported significant associations with depression, although the direction of associations was inconsistent.^19, 42, 43, 44^ A small case–control Japanese study (*n*=38) found that *BDNF* promoter I was hypomethylated in blood of severely depressed patients (mean age 45 years), with methylation differences varying from <0.1% to 56% depending on the CpG unit.^43^ However, they investigated a different *BDNF* region compared with our study, using different tissues and populations (clinical setting, age and ethnicity), which could account for the differences observed. Another study with blood cells reported significant hypermethylation in depressed patients in promoter I, with an effect size of 8%.^42^ The participants (*n*=85) were age-matched and on stable medication, but further characteristics were not given. A study of Japanese adults (20–60 years, *n*=180) found that depressed individuals had up to 4.6% lower promoter I methylation in saliva,^44^ as well as a negative association with methylation across the whole gene region. However, the investigators used the Kessler-6 Scale, a self-rated non-specific psychological distress scale that does not accurately diagnose depression.^44^ Another study using saliva (*n*=190) found no significant association but it was focused on depression in maltreated children, which may have a different pathophysiology from geriatric depression.^45^

Four studies have investigated promoter IV methylation, all of them using blood tissue. In line with our results, one study using a Korean post-stroke depression (*n*=244) cohort found that higher methylation was associated with depression.^19^ This was supported by another study that showed that among 732 Korean elderly (age⩾65 years), higher promoter IV methylation was associated with depression prevalence (*n*=101) and 2-year incidence (*n*=86). They did not consider medication use.^18^ By contrast, Fuchikami *et al.*^43^ did not find significant associations but their study (*n*=38) was underpowered to detect small effect sizes and differed from ours in terms of tissue type and population ethnicity. Tadić *et al.*^32^ found significant associations between higher (~1%) promoter IV methylation in leukocytes and better antidepressant response (*n*=39).

### Interaction between genetic and epigenetic variation at BDNF

Our study is among the first to examine the potential effects of *BDNF* genotype on modifying the association between depression and *BDNF* methylation levels. Three polymorphisms, including the widely investigated Val66Met (*rs6265*), were found to modify the association, such that for minor allele carriers of *rs6265* and *rs7103411* and major allele carriers of *rs908867*, depression was specifically associated with elevated *BDNF* methylation (Supplementary Figure S5). Although there is no clear evidence linking these variants with depression in our study, *rs6265* Met allele carriers have been shown to have an increased risk of suicide.^46, 47^ In a post-mortem study, *BDNF* was found to be hypermethylated in the brain of suicide completers.^48^ Furthermore, neuroimaging and stress exposure studies suggest that carriers of the Met allele have impaired fear, stress and anxiety regulation systems, making them more susceptible to depression.^49, 50, 51^ These findings align with our results demonstrating increased *BDNF* promoter methylation in moderate-to-severely depressed individuals who carry the *rs6265* minor allele.

In contrast to our results, two Korean studies by the same group found no significant interaction between promoter IV methylation in blood, *rs6265* genotype (the only variant examined) and depression.^18, 19^ However, one of the studies investigated the aetiologically different post-stroke depression.^19^ Furthermore, differences in the frequency of the *rs6265* minor Met allele across ethnic populations, is highly likely to account for the divergent findings. Indeed, in our Caucasian population there were only 4.1% of participants who were homozygous for the Met allele, which contrasts starkly with the 19.4% observed in the Korean study. Such differences have also been reported previously between Croatian and Korean populations (Met/Met frequency 3.4% versus 23.4%, respectively).^52^ The mechanisms by which SNPs interact with the epigenome to modulate psychiatric disorders remain largely unknown, and even in the broader molecular context removed from a given phenotype, the relationship between genetic variation and DNA methylation remains to be fully elucidated. Among commonly hypothesised mechanisms, genetic variants could influence the probability of DNA methylation and the location of a SNP may affect how it interacts with the epigenome or phenotype. DNA methylation can modulate the expression of genes, thus potentially augmenting or diminishing effects driven by individual genetic variants. *Rs6265* is in a protein-coding region of the gene, may alter BDNF protein function and one study reported that the Met allele was associated with increased protein concentrations.^53^ However, polymorphisms in other regions, including promoter or intronic regions, have also been shown to affect gene regulation, demonstrating that physical proximity is not essential.^54, 55^ Further investigation of the role of *BDNF* genetic variation in influencing the association between methylation levels and depression (Supplementary Figure S5) is required.

### Strengths and weaknesses

Our study is one of the largest in this field to date, with a sample size of over 1000, allowing more power to detect smaller methylation differences. Unlike most previous studies, we were thus able to consider a range of potential confounding factors linked to both depression status and methylation levels. This is also one of the first studies to consider both genetic and epigenetic variation in depression. One limitation of our study is that we assessed buccal samples collected at follow-up, ~8 years after baseline depression was assessed. However, late-life depression is often a chronic disorder^56^ and participants with depression at baseline were also highly likely to have depression over follow-up, and thus at the time buccal samples were collected. Indeed, in our study there was a very strong correlation between baseline depression and chronic depression over follow-up (*P*<0.0001). Furthermore, chronic depression was also significantly associated with *BDNF* methylation at the same CpG sites (Supplementary Table S4), with associations being even stronger than with baseline depression. This suggests that methylation differences may be a stable marker of depression. However, future studies should aim to assess both phenotype and DNA methylation longitudinally to investigate associations over time.

We should also consider the small effect sizes observed as we do not yet know how these could translate into biological differences. However, the cumulative effects of such small changes to the epigenome over a long period of time, or in multiple genes in the same biological pathway, might be anticipated to result in phenotypic differences large enough to cross a disease ‘threshold'. Other studies in epigenetic psychiatry have also reported small but significant effect sizes,^32, 43, 44, 57, 58^ supporting our observations. No adjustment for multiple comparisons was made, and only the association between *BDNF* promoter I methylation at CpG unit 3.4.5 would remain significant at the Bonferroni corrected level of *P*=0.0028 (that is, for 18 tests). However, Bonferroni correction would result in an inflated type-2 error rate, especially given the assumption of independent tests that does not hold true for methylation levels at individual CpG sites, which are correlated.

It remains unclear whether methylation is a driver or a consequence of depression, or a combination of both. Plasma BDNF levels have been found to predict disease outcomes of MDD patients,^8^ and findings from our study suggest *BDNF* hypermethylation in prevalent and chronic depression. But no study has yet examined changes in *BDNF* methylation levels over time. Establishing causation is important to understand the function of disease-associated epigenetic marks, furthering knowledge on the aetiology of the disease, as well as the identification of diagnostic and therapeutic tools.^59^ Thus, longitudinal studies with biospecimens collected early in life prior to disease onset and followed-up at multiple time points throughout disease progression or recovery are now needed.

### Towards epigenetic biomarkers for depression

One of the keys aims in behavioural epigenetic studies is the search for peripheral biomarkers of psychiatric disease. Although few studies have directly investigated the relationship between brain and peripheral epigenetic landscapes, peripheral biomarkers are important because brain tissue cannot be easily extracted, rendering brain-based biomarkers inconvenient. Psychiatric disorders have also been shown to involve systemic effects.^60^ Previous research has made a case for *BDNF* methylation in blood as a biomarker for depression.^30, 43^ However, buccal tissue may prove more informative as a surrogate tissue than blood.^61^ Unlike blood that is of mesodermal origin, buccal tissue has the same germ cell layer of origin (that is, ectodermal) as neural tissue, and thus has been speculated to be a more relevant peripheral tissue for epigenetic analysis in psychiatric disorders.^62^ Buccal biomarkers also confer several advantages to blood-based markers, including being less invasive,^63^ and buccal cells are a more uniform cell population, reducing the problematic issue of cell heterogeneity in epigenetic studies.^64^ In addition to significant differences in individual methylation sites, our findings highlight a general pattern of *BDNF* hypermethylation in the buccal tissue of depressed individuals. More research is needed to determine the true discriminatory potential of methylation as a biomarker for depression, especially given the small effect sizes observed.

## Conclusion

As one of the largest studies investigating methylation in depression, our findings add further support for the role of differential *BDNF* methylation, and suggest that genetic variation in *BDNF* mediates these associations. Our findings thus highlight the potential for *BDNF* methylation in buccal tissue to be a biomarker of depression, but further large prospective longitudinal studies are needed to confirm our findings and reveal the temporal relationship of the observed associations.

## Figures and Tables

**Table 1:** Sample characteristics according to depression status at study inclusion^a^

*Characteristic*	*No depression*	*Depression*	P*-value*^b^
*n*	773	251	—
Age (mean±s.d.)	71.4±4.5	72.0±4.5	0.045
Proportion of women (%)	55	77	<0.001

*Proportion (%) of individuals who are or have:*			
High education level^c^	41.4	30.7	0.002
Living alone	20.1	36.7	<0.001
Habitual alcohol drinkers^d^	19.8	14.4	0.061
Habitual smokers^e^	39.0	33.3	0.108
Functional impairment^f^	1.3	4.0	0.007
Hypertension^g^	43.5	46.6	0.383
Hypercholesterolaemia^h^	32.2	30.8	0.687
Ischaemic disease^i^	10.5	10.4	0.957
Obesity^j^	7.7	9.2	0.434
Diabetes^k^	7.2	5.6	0.386
Thyroid disease	6.5	9.2	0.145
Comorbidities^l^	13.3	12.0	0.574
Cognitive impairment^m^	4.2	12.8	<0.001

*Proportion (%) of individuals using antidepressants:*			
TCA^n^	0.8	2.4	0.039
SSRI^o^	1.0	4.0	0.002
Other	0.4	2.8	0.001

Abbreviations: ADL, Activities of Daily Living scale; CES-D, Centre of Epidemiological Studies Depression; IADL, Instrumental Activities of Daily Living scale; MDD, major depressive disorder; MMSE, Mini-Mental State Examination score.

a Current MDD or CES-D ⩾16.

b On the basis of a *χ*^2^-test (except age, for which a Student's *t*-test was used).

c Undergone post-secondary education of any type.

d More than 24 g of alcohol per day.

e More than 10 pack-years (number of packs per day × years smoked).

f Unable to independently complete two items on both or either of the IADL scale items and the ADL scale.

g Resting blood pressure ⩾160/95 mmHg or reported treatment.

h Total cholesterol ⩾6.2 mmol l^−1^ or treated.

i History of cardiovascular disease (for example, angina pectoris, myocardial infarction, stroke, cardiovascular surgery and arteritis).

j Body mass index ⩾30 kg/m^2^.

k Fasting glucose ⩾7.0 mmol l^−1^ or reported treatment.

l Having a history of cardiovascular diseases (for example, angina pectoris, myocardial infarction, stroke, cardiovascular surgery and arteritis), more than one chronic illnesses (high blood pressure, high cholesterol, diabetes, thyroid problems and asthma) or cancer diagnosed within the last 2 years.

m MMSE score <24.

n Tricyclic antidepressants.

o Selective serotonin reuptake inhibitors.

**Table 2:** Percentage of participants with specific *BDNF* genotypes in the study population

*Polymorphism*^a^	*Alleles*		*Percentage of total participants (%)*						P*-value*^b^
	*Major (M)*	*Minor (m)*	*No depression*			*Depression*			
			*MM*	*Mm*	*mm*	*MM*	*Mm*	*mm*	
*rs6265*	G	A	45.8	25.4	3.5	15.4	9.0	0.9	0.126
*rs908867*	G	A	62.2	11.0	0.4	20.9	4.0	0.1	0.905
*rs962369*	A	G	39.2	26.6	5.0	14.2	8.4	1.9	0.648
*rs1491850*	T	C	24.1	34.7	14.1	7.7	13.3	3.6	0.136
*rs7103411*	T	C	42.8	27.5	4.2	14.5	9.4	1.0	0.696
*rs11030101*	A	T	21.5	36.0	16.0	6.2	13.3	5.0	0.351
*rs28722151*	C	G	23.8	35.5	14.2	7.3	13.2	4.2	0.391

a Locations of polymorphisms are presented in Supplementary Figure S1.

b *χ*^2^-tests were used to calculate *P-*value.

## Supplementary Material

Supplementary Informations
