# Supplementary material for: BDNF promoter methylation and genetic variation in late-life depression
Source: Transl Psychiatry. 2015 Aug 18;5(8):e619–. doi: 10.1038/tp.2015.114 (PMC4564567; doi:10.1038/tp.2015.114)
Supplement: Supplementary Informations [file tp2015114x1.doc]

**
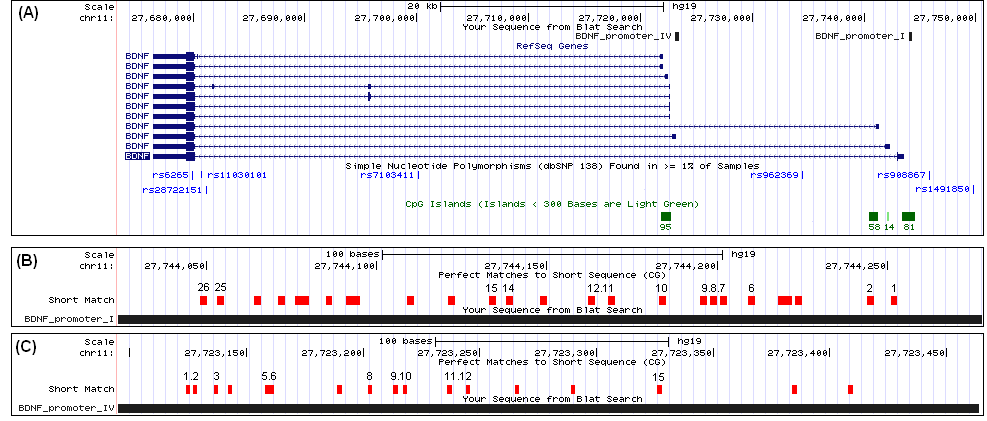
**

**Figure S1.** UCSC genome browser [http://ucsc.genome.edu] view of the *BDNF* gene and SEQUENOM assays. (A) *BDNF* gene on chromosome 11. The direction of transcription is indicated by arrows. Solid dark blue bars denote exons, and arrowed lines denote introns. Several splice variants are shown. Locations of relevant SNPs (*rs6265, rs11030101, rs28722151, rs7103411, rs962369, rs908867, rs1491850*) are shown in bright blue. Regions of interest in the previously studied promoter I and promoter IV are indicated and denoted by black bars above the gene structure. Green bars denote CpG islands, and the number of CpG sites within each island is indicated below. Magnified images of regions of interest in promoters I (B) and IV (C) are shown. Individual CpG sites detected by each assay are highlighted in red. CpG sites are numbered according to the predicted cleavage pattern from SEQUENOM MassARRAY. Detectable sites are numbered.

(A)


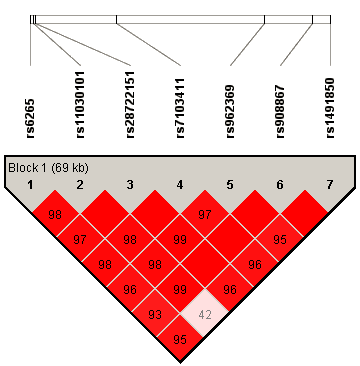


(B)


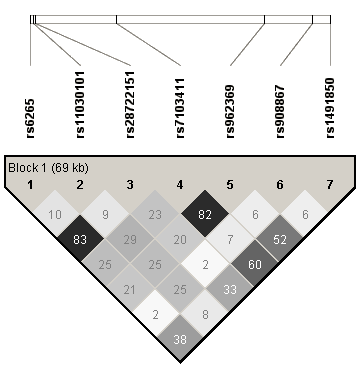


**Figure S2.** Linkage disequilibrium map of the seven SNPs investigated across *BDNF* using D’ (A) and r2 (B). The darker the square the higher the correlation.


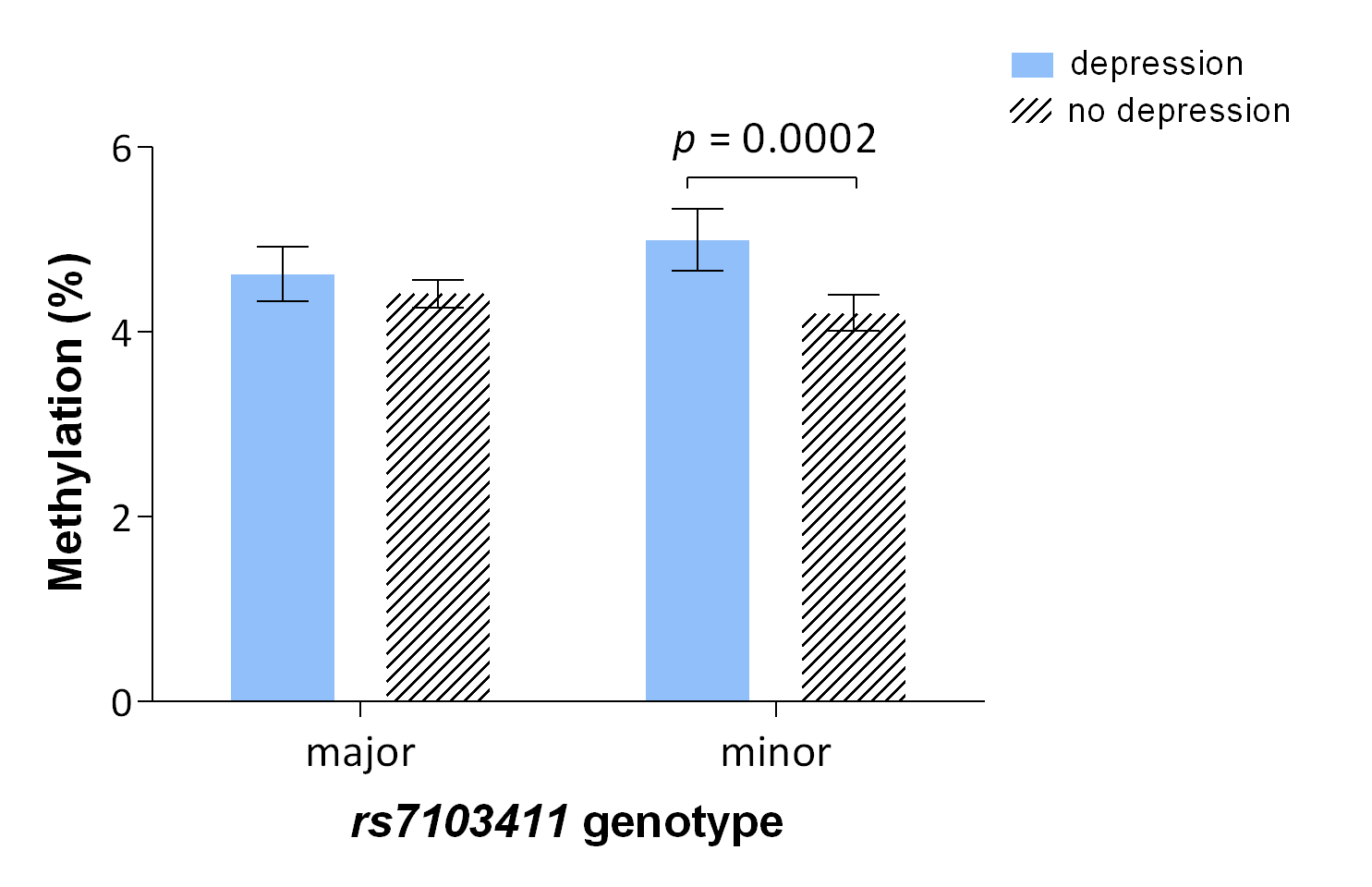


**Figure S3.** Comparison of *BDNF* promoter I methylation at CpG unit 3.4.5 in depressed and non-depressed individuals, stratified according to the presence of *rs7103411* minor allele. Data is presented as the geometric mean methylation (%) ± 95% CI. P-values were calculated using student’s t-test (n=507 major homozygotes, 403 heterozygotes and minor homozygotes).


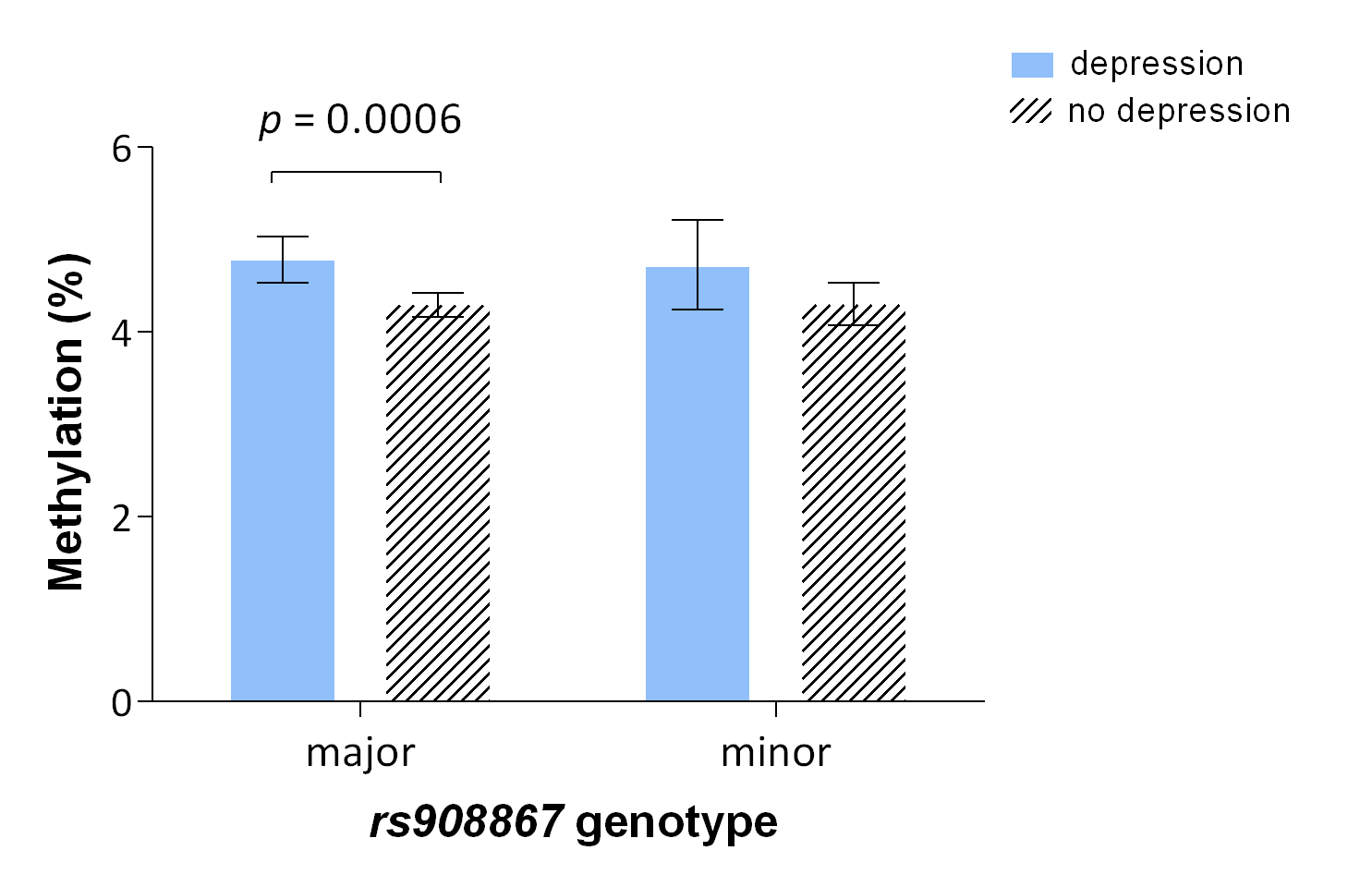


**Figure S4.** Comparison of *BDNF* promoter I methylation at CpG unit 3.4.5 in depressed and non-depressed individuals, stratified according to the presence of *rs908867* minor allele. Data is presented as the geometric mean methylation (%) ± 95% CI. P-values were calculated using student’s t-test (n=794 major homozygotes, 148 heterozygotes and minor homozygotes)

**
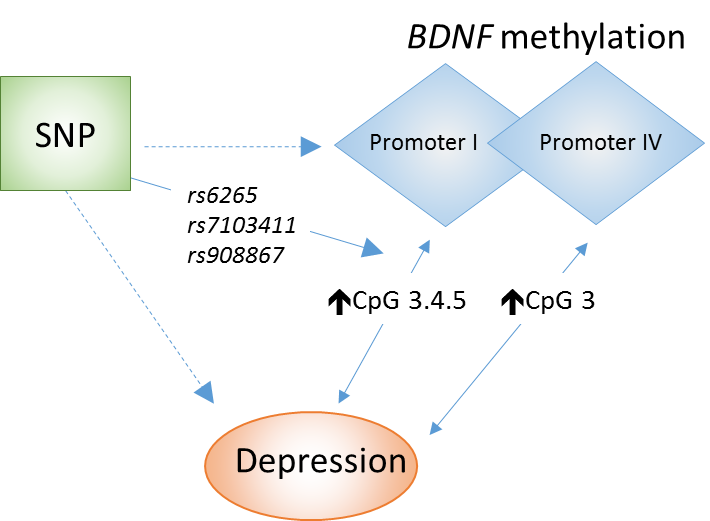
**

**Figure S5.** A schematic representation of the interaction between SNPs, *BDNF* methylation and depression. Solid lines indicate significant associations that were found in this study; dotted lines indicate potential associations that were not identified here.

**Table S1**. Primer sequences and PCR cycling conditions

| ***BDNF* promoter** | **Amplicon length (bp)** | **Primers**  **(5' - 3')** |
| --- | --- | --- |
| I | 255 | Forward:  aggaagagagGGTAGAGGTAGGGAGATTTTATGTTAG  Reverse: cagtaatacgactcactatagggagaaggctCCTACCCCCACTCTAATTAAAACAA |
| IV | 372 | Forward:  aggaagagagGTAGGGTTTTTTGGGAGAGTTTTT  Reverse:  cagtaatacgactcactatagggagaaggctTTCCCAAATATAAATTAACAACCCC |

**Table S2.** Genomic sites of methylation interrogated in the current study.

| **Amplicon** | **Analytic Unit** | **Genomic location^a^** |
| --- | --- | --- |
| ***BDNF* promoter I** | CpG 1 | CpG11:27,744,252 |
|  | CpG 2 | CpG11:27,744,245 |
|  | CpG 3.4.5 | CpG11:27,744,224  CpG11:27,744,221  CpG11:27,744,219 |
|  | CpG 6 | CpG11:27,744,210 |
|  | CpG 7.8.9 | CpG11:27,744,202  CpG11:27,744,199  CpG11:27,744,196 |
|  | CpG 10 | CpG11:27,744,184 |
|  | CpG 11.12 | CpG11:27,744,169  CpG11:27,744,163 |
|  | CpG 14 | CpG11:27,744,139 |
|  | CpG 15 | CpG11:27,744,134 |
|  | CpG 25 | CpG11:27,744,054 |
|  | CpG 26 | CpG11:27,744,049 |
| ***BDNF* promoter IV** | CpG 1.2 | CpG11:27,723,125  CpG11:27,723,128 |
|  | CpG 3 | CpG11:27,723,137 |
|  | CpG 5.6 | CpG11:27,723,159  CpG11:27,723,161 |
|  | CpG 8 | CpG11:27,723,203 |
|  | CpG 9.10 | CpG11:27,723,214  CpG11:27,723,218 |
|  | CpG 11.12 | CpG11:27,723,237  CpG11:27,723,245 |
|  | CpG 15 | CpG11:27,723,327 |

^a^As per hg19 nomenclature (1).

**Table S3.** Cycling conditions for amplification PCR

| **Step** | **Cycles** | **Temperature (^o^C)** | **Time** |
| --- | --- | --- | --- |
| **1** | 1 | 95 | 10 min |
| **2** | 5 | 95  56  72 | 10 s  30 s  2 min |
| **3** | 40 | 95  60  72 | 10 s  30 s  1.5 min |
| **4** | 1 | 72 | 7 min |
| **5** | 1 | 4 | ∞ |

**Table S4.** Comparison of the association between *BDNF* methylation and baseline or chronic depression, in unadjusted analysis.

|  | **Baseline depression** | | **Chronic depression** | |
| --- | --- | --- | --- | --- |
|  | Δ mean (%) | p-value | Δ mean (%) | p-value |
| ***BDNF* promoter I** |  |  |  |  |
| **CpG 1** | 0.25 | 0.097 | 0.44 | 0.016 |
| **CpG 3.4.5** | 0.40 | 0.0002 | 0.44 | 0.0019 |
| **CpG 7.8.9** | 0.19 | 0.074 | 0.22 | 0.064 |
| ***BDNF* promoter IV** |  |  |  |  |
| **CpG 3** | 5.40 | 0.021 | 7.52 | 0.0061 |

**Table S5.** Linear regression models for the association between depression status and *BDNF* methylation levels.

| **CpG unit** | **unadjusted** | | **adjusted** | |
| --- | --- | --- | --- | --- |
|  | **β (SE)** | ***p*-value** | **β (SE)** | ***p*-value** |
|  |  |  |  |  |
| *BDNF* promoter I: 3.4.5 | 0.103 (0.027) | 0.0002 | 0.094 (0.029) ^a^ | 0.001 |
|  |  |  |  |  |
| *BDNF* promoter IV: 3 | 0.36 (0.16) | 0.021 | 0.24 (0.13) ^b^ | 0.067 |
|  |  |  |  |  |

^a^Adjusted for sex, age and antidepressant use.

^b^Adjusted for sex, age, antidepressant use and functional impairment.

**References**

1. Saffery R. and Gordon L. (2015): Is it time for a standardized system of reporting for sites of genomic methylation? *Genome Biology* *in press*.
